# Reminder: Interpret the “Science” with Caution

**DOI:** 10.5704/MOJ.2007.027

**Published:** 2020-07

**Authors:** RY Kow, CL Low

**Affiliations:** 1 Department of Orthopaedics, International Islamic University Malaysia, Kuantan, Malaysia; 2 Department of Radiology, Hospital Tengku Ampuan Afzan, Kuantan, Malaysia

Dear Editor,

As the SARS-CoV-2 virus-induced Covid-19 sweeps through the whole world like a wildfire, crumbling most of the economy along the way with no proven vaccine or cure available, scientists and clinicians alike are scrambling to find the cure for this debilitating disease. As everyone jumps into the bandwagon of Covid-19 research, publications on their findings and opinions follow. Almost all of the medical journals, respiratory medicine-related or otherwise, rush to publish Covid-19-related articles, understandably as Covid-19 not only directly infects and kills patients at risk, it also causes delayed or subpar management of other patients. As a result, excess all-cause mortality is currently touted as a better gauge of the medical, societal, and economic burden for Covid-191.

In order to publish the latest findings on Covid-19 in the shortest period, journals have opted to expedite the review process while some journals even publish manuscripts without undergoing peer review, potentially sowing misinformation and misguidance. The most notable example is the use of hydroxychloroquine for the treatment of Covid-19, which has resulted in much confusion among clinicians, especially when confronted by the next-of-kin of infected patients. While it is noble to offer a glimpse of hope in curing Covid-19, it is immoral to skew and manipulate people’s perceptions to derive desired conclusions using dubious data. Even the most prestigious journals like Lancet and New England Journal of Medicine are not spared from these blushes^2^. To their credit, they offer timely expression of concern in these published articles. To date, in regard to Covid-19-related articles, there are 13 retracted articles, 2 temporarily retracted articles and 3 articles with expression of concern^2^. To make matter even worse, those articles with errors or misinformation may persist for a long duration prior to retraction, with an estimated lag-time of three years^3^. As in the case of hydroxychloroquine, although some researchers have blown the whistle on the danger of following the trend blindly without any randomised controlled trial, it does not deter people from championing it as the savior for all.

From an orthopaedic point of view, the “discovery” of these new Covid-19 treatments does not directly change the way we are practicing, as we are not directly involved in the clinical trials or actively using these drugs to treat patients. Nevertheless, it serves as a reminder that we should critically appraise all publications, especially those articles which have not gone through reviewing processes. We might be doing more harm than good, albeit with a good intention, if we blindly follow the trend. In terms of journal, it remains as a rule of thumb to “review, review and review” to maintain the integrity and standard of published articles. On this note, we should be thankful to all peer reviewers who have volunteered their precious time to review and provide constructive comments for the betterment of mankind knowledge.
